# Pancreatic cancer in Saudi Arabia (2005–2020): increasing trend

**DOI:** 10.1186/s12885-024-12401-8

**Published:** 2024-05-29

**Authors:** Nasr Eldin Elwali, Saad Mohammed AlShareef, Ammar H. Khamis, Moawia M. A. Elhassan

**Affiliations:** 1 Deanship of Scientific Research, Imam Mohammad Ibn Saud Islamic University (IMSIU), Riyadh, Saudi Arabia; 2 Department of Medicine, Imam Mohammad Ibn Saud Islamic University (IMSIU) College of Medicine, Riyadh, Saudi Arabia; 3 Mohammed Bin Rashid University of Medicine and Health Sciences, Dubai, United Arab Emirates; 4 Department of Oncology, University of Gezira, National Cancer Institute, Wad Medani, Sudan

**Keywords:** Pancreatic cancer, Saudi Arabia

## Abstract

Pancreatic cancer, a highly fatal malignancy, has shown a global rise in the incidence and mortality rates. However, these rates vary significantly across different regions worldwide. This study aims to assess the incidence and mortality of pancreatic cancer in Saudi Arabia. We collected the data from 16 annual cancer incidence reports in Saudi Arabia for the study period (2005–2020) and from the WHO's IARC Global Cancer Observatory website. Although the burden of pancreatic cancer in Saudi Arabia is relatively lower compared to global rates, the disease incidence has shown a steady increase over the study period, in addition to regional variations within the country. The disease predominantly affects the elderly population, aged 50 years and above in both genders, with males exhibiting higher rates than females. Further studies are required to identify the potential risk factors for pancreatic cancer in the Saudi population.

## Introduction

Globally, pancreatic cancer ranks as the 12th most common cancer and the 7th cause of cancer death [1, 2]. It has a high mortality rate, with a 5-year overall survival rate of only about 10% [1]. The incidence and mortality of pancreatic cancer varies widely worldwide [3]. The global variations in incidence and mortality rates were linked to the human development index (HDI); according to the classification of the World Bank, with High-Income Countries (HICs) generally have higher age-standardized incidence and mortality rates than Low- and Middle-Income Countries (LMICs) [4, 5]. Over the past few decades, the global burden of pancreatic cancer has more than doubled [6].

The causes of pancreatic cancer are not fully understood; however, identified risk factors include both genetic and environmental elements such as smoking, alcohol intake, obesity, physical inactivity, diet quality, diabetes, chronic pancreatitis, and peptic ulcer [7–10]. Smoking, alcohol intake, and chronic pancreatitis are considered as high-risk factors for pancreatic cancer [11]. A better diet quality, i.e., a dietary pattern that involves adequate intake of fruits, vegetables, and fiber compared to the starch-rich and western dietary patterns, has been associated with reduced risk of pancreatic cancer [12]. Pancreatic cancer affects males more than females, a variation that is common in both cancer incidence and mortality, which may be attributed to differences in sex hormones and genetic and epigenetic factors [13]. Genetic variations, such as *BRCA1/2*, are linked to increased susceptibility to pancreatic cancer [14]. Identifying these genetic variants could lead to recognizing high-risk patients, facilitating prompt screening and early disease diagnosis [6]. Familial pancreatic cancer is defined as a condition where at least two first-degree family members are affected by the disease without evidence of being part of cancer syndrome. It was found that up to 20% of pancreatic cancer patients have a family history of the disease [15].

Worldwide, most pancreatic cancer patients are diagnosed at later stages with advanced unresectable tumors. This is also the case in Saudi Arabia, where patients with pancreatic cancer are typically diagnosed at advanced stages. The surveillance of pancreatic cancer—as a cancer of low incidence and high mortality—has focused on the high-risk groups but not on the general population [16]. Therefore, screening would lead to early disease detection, improving the possibility for a better prognosis and treatment outcome1 [17–19]. Nevertheless, the disease is associated with high mortality rates due to insufficient screening methodologies that result in late diagnosis of advanced stages that hinder the efforts of effective treatment of the disease [20].

Several organizations have published guidelines for pancreatic cancer screening, particularly for individuals at a high genetic risk [21]. However, in Saudi Arabia, there are still neither guidelines nor recommendations for pancreatic cancer screening.

In Saudi Arabia, there has been a general increase in cancer incidence, which was partially attributed to the changes in the population's lifestyle due to the improvement in the socioeconomic status as a consequence of the Kingdom's flourishing economy [22]. However, there is a scarcity of studies exploring the epidemiology of pancreatic cancer in Saudi Arabia. In an epidemiological study revealed a general increase in cancer incidence in Saudi Arabia between 1990 and 2016, specifically, pancreatic cancer showed a fourfold increase, other types of cancer demonstrated varying rates of increase: thyroid and breast cancer incidences surged 26-fold and tenfold, respectively, while liver cancer and lymphoma exhibited more modest increases of threefold and twofold, respectively [22]. Regional variations in cancer incidence across different administrative regions of Saudi Arabia have been reported in previous studies [23, 24].

The limited availability of data on survival and mortality rates for pancreatic cancer in certain regions, including Saudi Arabia, makes it difficult to fully comprehend the actual patterns of this disease and its impact on different areas. A recent bibliometric analysis of pancreatic cancer research identified significant gaps and limitations in Middle Eastern and North African countries [25]. These data gaps pose a significant challenge for policymakers at both regional and national levels. This study aims to shed light on the current burden of pancreatic cancer in Saudi Arabia, specifically in terms of incidence and mortality rates.

## Methods

Data on pancreatic cancer incidence were collected from 16 annual cancer incidence reports in Saudi Arabia for the study period (2005–2020). World pancreatic cancer incidence and mortality data were collected from the World Health Organization (WHO), the International Agency for Research on Cancer (IARC), Global Cancer Observatory website [26].

The Saudi cancer registry is a population-based registry managed and operated by the Saudi Ministry of Health through the Saudi Heath Council. It collects data from all over the country through five regional offices to ensure complete coverage of the country's healthcare facilities. The registry publishes the annual cancer incidence reports in Saudi Arabia [27].

As mentioned above all the data analyzed in this study were collected from the following two publicly available data sources which were accessed in December 2023:

1. Annual reports of the Saudi Cancer Registry. 
https://shc.gov.sa/Arabic/NCC/Activities/Pages/AnnualReports.aspx.
2. WHO, International Agency for Research on Cancer, Global Cancer observatory website.
https://gco.iarc.fr/en.

### Statistical analysis

The data were analyzed using the software IBM-SPSS for Windows version 29.0 (SPSS Inc., Chicago, IL). Continuous variables were described using measures of central tendency and measures of dispersion. The Kolmogorov–Smirnov test was used to assess the normality of continuous variables, such as the incidence rate. A two-independent t-test was used to compare the incidence rate between males and females. Linear regression was used to predict the rate of incidence over time. A *P*-value of less than 0.05 was considered significant in all statistical analyses.

## Results

### Pancreatic cancer incidence

The incidence of pancreatic cancer is on the rise worldwide. According to IARC reports, i.e., the Global Cancer Observatory website, there were approximately half a million cases diagnosed in 2020, ranking 11th in cancer incidence, with an Age-Standardized Rate (ASR) of 4.9. The ASRs for males and females were 5.7 and 4.1, respectively. It ranks 12th for males and 11th for females (Table 1) as a common cancer.

**Table 1:** Estimated number of pancreatic cancer cases in 2020, world, both sexes, all ages (WHO, IARC Global Cancer Observatory Website, 2020)

	**New cases**				**Deaths**			
	Number	Rank	Age Standardized Incidence Rate (ASR)^a^	Cumulative Risk^b^	Number	Rank	Age Standardized Incidence Rate (ASR)	Cumulative Risk
Both Sexes	495,773	11	4.9	1.63	466,003	7	4.5	1.58
Males	262,865	12	5.7	1.8	246,840	7	5.3	1.76
Females	232,908	11	4.1	1.48	219,163	7	3.87	1.43

^a^The age-standardized rate is a summary measure of the of cancer incidence rate that a population would have if it had a standard age structure. The rate is expressed per 100.000 population

^b^The cumulative risk is the risk that an individual would have of developing the cancer in question during a certain age span if no other causes of death were in operation

### World regional variation in pancreatic cancer incidence

Worldwide, there is a wide variation in the incidence of pancreatic cancer (Table 2). Northern America has the highest ASR for both sexes (8.0), followed by Europe (7.8) and Oceania (6.6). Africa has the lowest ASR for both sexes (2.3). Among males; Europe also has the highest ASR (9.4), followed by Northern America (9.3) and Oceania (7.3). Among females; Europe and North America have the highest ASR (6.4), followed by Oceania (6.0) while Asian and African females reported the lowest ASR 3.3 and 2.0, respectively.

**Table 2:** Estimated number of new cases and deaths of pancreatic cancer in 2020, worldwide, all ages (WHO, IARC Global Cancer Observatory Website, 2020)

	**New cases**				**Deaths**		
		Number of cases	Rank	Age Standardized Incidence Rate (ASR)	Number of cases	Rank	Age Standardized Incidence Rate (ASR)
Asia	Both Sexes	233,701	12	4.0	224034	7	3.8
	Males	129,488	11	4.7	123337	6	4.5
	Females	104,213	12	3.3	100697	9	3.1
Europe	Both Sexes	140,116	7	7.8	132134	4	7.2
	Males	70,210	8	9.4	66698	4	8.8
	Females	69,906	6	6.4	65436	4	3.1
Northern America	Both Sexes	62,643	11	8.0	53277	3	6.5
	Males	32,938	9	9.3	27888	4	7.6
	Females	29,705	8	6.4	25389	4	5.5
Latin America and the Caribbean	Both Sexes	37,352	11	4.5	36030	7	4.3
	Males	18,477	10	5.0	17897	6	4.9
	Females	18,875	9	4.0	18133	6	3.8
Africa	Both Sexes	17,070	17	2.3	16549	13	2.3
	Males	9239	13	2.7	8936	10	2.6
	Females	7831	17	2.0	7613	11	1.9
Oceania	Both Sexes	4891	10	6.6	3979	5	5.2
	Males	2513	11	7.3	2084	5	6.0
	Females	2378	9	6.0	1895	4	4.5

### Pancreatic cancer incidence in the Gulf Cooperation Council countries

The Gulf Cooperation Council (GCC) includes Saudi Arabia, Kuwait, Qatar, Bahrain, the United Arab Emirates (UAE), and the Sultanate of Oman. The number of new cases of pancreatic cancer for both sexes reported in 2020 was 568 for Saudi Arabia, 107 for Kuwait, 97 for Oman, 80 for the United Arab Emirates, and 33 and 32 for Bahrain and Qatar, respectively (Table 3). Pancreatic cancer incidence ASR was (4.2) for Kuwait, while the lowest pancreatic cancer incidence among GCC countries, ASR (2.2) was reported for Saudi Arabia (Table 3).

**Table 3:** Pancreatic cancer in the GCC countries (WHO, IARC Global Cancer Observatory Website, 2020)

	New cases			Deaths			5-year survival	
	Number of cases	Age Standardized Incidence Rate (ASR)	Rank	Number of cases	Age Standardized Mortality Rate (ASR)	Rank	Number of cases	Prop. (per 100 000)
Saudi Arabia	568	2.2	16	559	2.2	8	544	1.56
Bahrain	33	3.5	14	30	3.3	8	30	1.76
Kuwait	107	4.2	12	105	4.3	6	90	2.11
Qatar	32	3.4	15	30	3.3	9	28	0.97
Oman	97	2.6	16	77	2.7	9	79	1.55
United Arab Emirates	80	2.4	19	77	2.3	7	74	0.75

### Pancreatic cancer incidence in Saudi Arabia

**Fig. 1:**
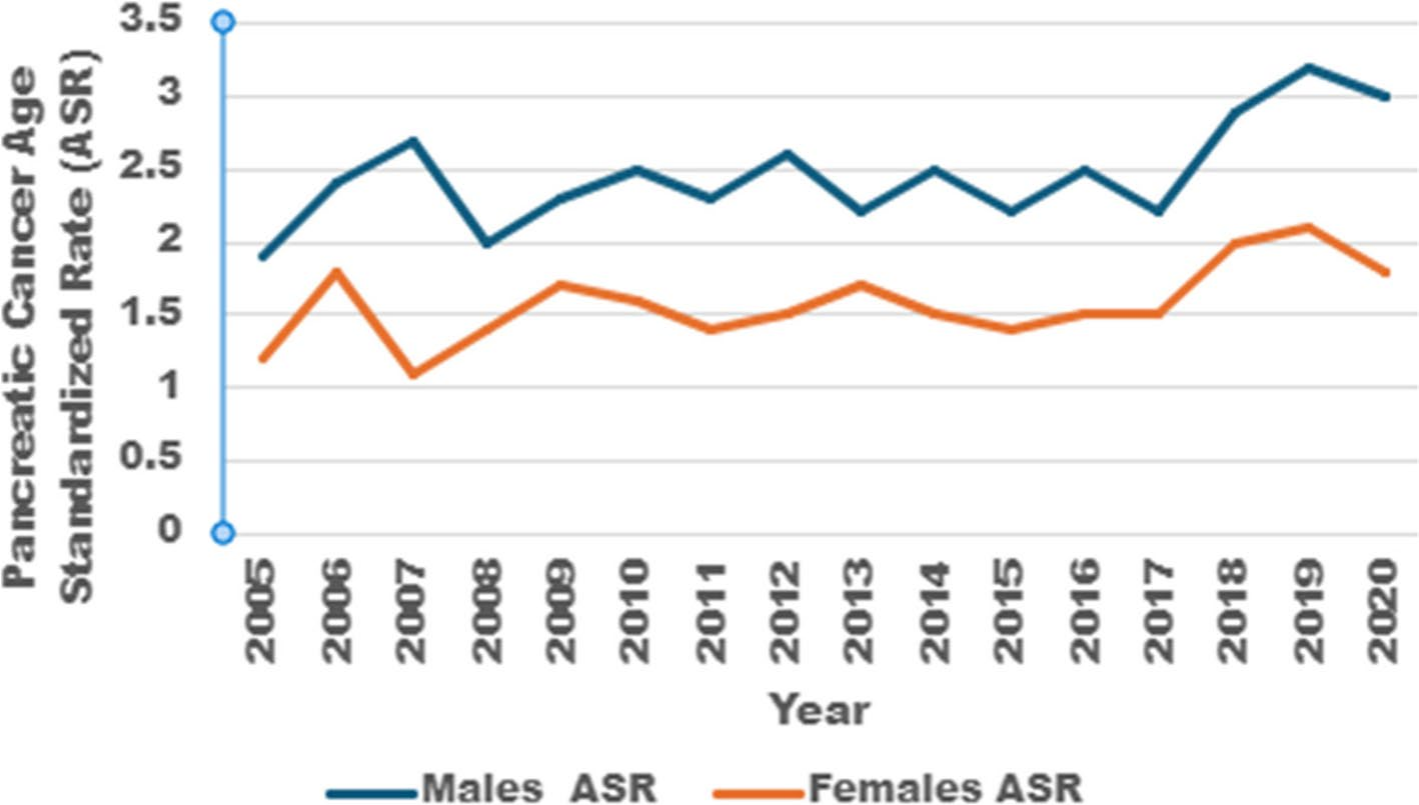
.

In Saudi Arabia, pancreatic cancer incidence has been on the rise for the study period from 2005 to 2020 (Saudi Annual Cancer Incidence Reports). The number of new cases has increased from 131 in 2005 to 357 in 2020. Likewise, ASR also showed an increase in the same period, rising from 1.9 for males in 2005 to 3.0 in 2020, while for females, the increase was from 1.2 to 1.8 over the same period. (Table 4, Fig. 1). Although the incidence of pancreatic cancer in the Saudi population has been zigzagging, there has been a net increase in the last five years of the study period. When ANOVA regression analysis was done using pancreatic cancer incidence as the dependent variable, and year as an independent variable, it was shown that pancreatic cancer incidence increases with time, and that 37.2% of male cases and 32.7% of female cases can be explained by the year (*p* = 0.012 and 0.021 respectively).

**Table 4:** Pancreatic cancer incidence in Saudi Arabia (2005–2020), (Saudi Cancer Incidence Reports)

Year	**Males**		**Females**	
	Number of cases	Age Standardized Incidence Rate (ASR)	Number of cases	Age Standardized Incidence Rate (ASR)
2005	82	1.9	49	1.2
2006	93	2.4	73	1.8
2007	127	2.7	51	1.1
2008	100	2.0	67	1.4
2009	117	2.3	82	1.7
2010	114	2.5	81	1.6
2011	128	2.3	77	1.4
2012	155	2.6	87	1.5
2013	130	2.2	103	1.7
2014	175	2.5	102	1.5
2015	161	2.2	96	1.4
2016	170	2.5	104	1.5
2017	158	2.2	107	1.5
2018	209	2.9	141	2.0
2019	238	3.2	156	2.1
2020	226	3.0	131	1.8

In 2020, pancreatic cancer was the 9th most common cancer in males, with a frequency of 3.9% (Table 5). The total number of new pancreatic cancer cases diagnosed in 2020 was 357, which includes 226 (63.3%) males and 131 (36.7%) females (Table 4), with male to female ratio of 1.7:1. The mean values for pancreatic cancer ASR over the 16 years study period ± Standard Error of the Mean were 2.46 ± 0.089 and 1.58 ± 0.067 for males and females respectively, with statistically significant (*p* < 0.001) difference when t-test was applied. Most new cases (104 patients, 30%) occurred in the Riyadh Region, followed by the Makkah region (83 patients, 24%) and Eastern Region (70 patients, 20%).

**Table 5:** Top ten cancers reported among Saudi adults by gender (Saudi Cancer Incidence Report, 2020)

Males	Number of cases	%	Females	Number of cases	%
Colorectal	956	16.7	Breast	2444	32.7
NHL	387	6.8	Thyroid	808	10.8
Prostate	362	6.3	Colorectal	754	10.1
Leukemia	353	6.2	Corpus Uteri	494	6.6
Lung	349	6.1	NHL	322	4.2
Liver	285	5.0	Leukemia	246	3.3
Bladder	285	5.0	Ovary	233	3.0
Hodgkin’s Lymphoma	266	4.7	Hodgkin's Lymphoma	185	2.5
Pancreas	255	3.9	Brain, CNS	160	2.1
Thyroid	224	3.9	Liver	155	2.1

**Fig. 2:**
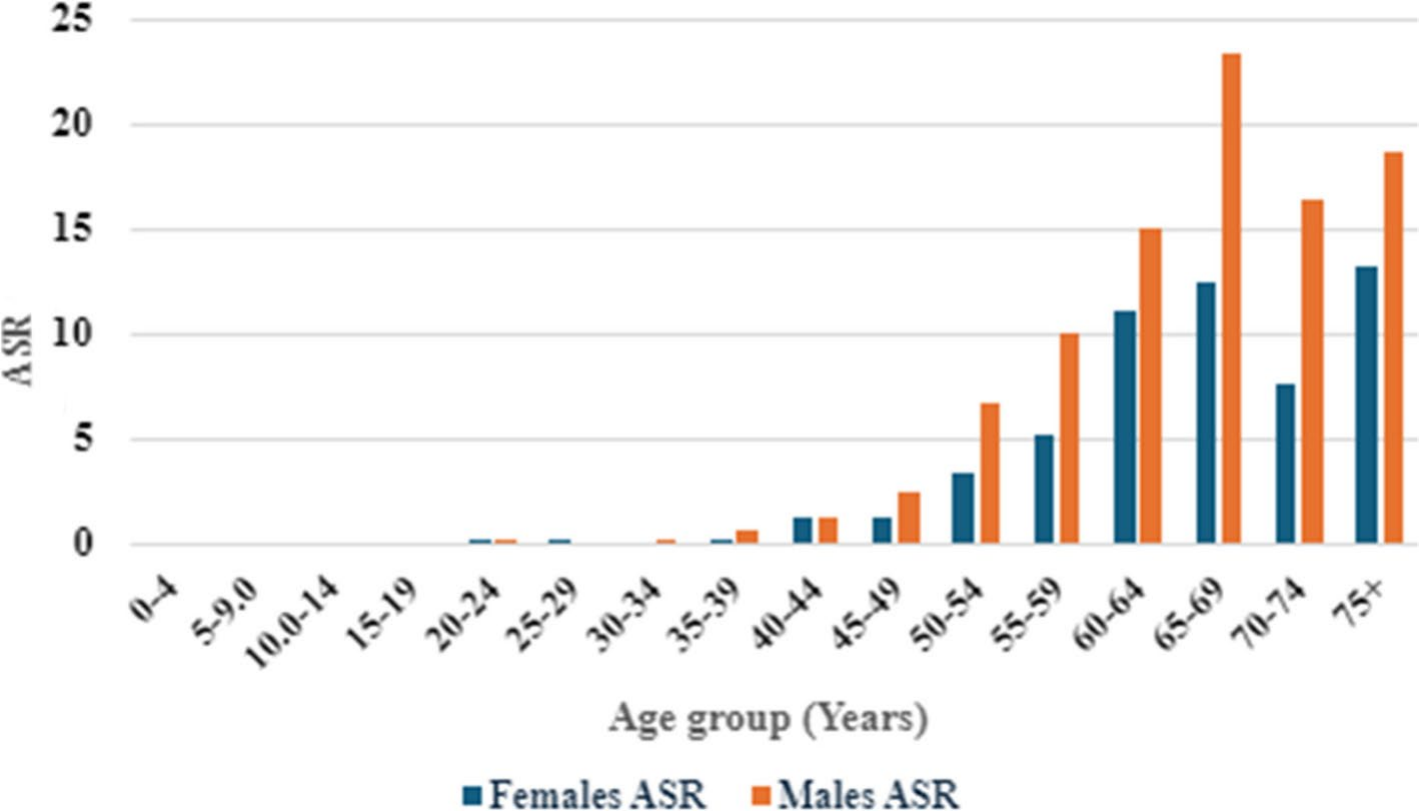
Age Standardized Incidence Rate (ASR) of pancreatic cancer by age groups among Saudi Males and females (Saudi Cancer Incidence Report 2020)

Pancreatic cancer in Saudi Arabia affects older patients, with more than 85% of the cases diagnosed in patients > 50 years old, both in males and females. The age group with the highest number of pancreatic cancer cases was (60–64 years) both in males and females. No pediatric pancreatic cancer cases were reported in 2020 (age < 19 years) (Cancer incidence report in Saudi Arabia 2020) (Table 6, Fig. 2).

**Table 6:** Age Standardized Incidence Rate (ASR) of pancreatic cancer by age groups among Saudi Males and females (Saudi Cancer Incidence Report 2020)

Age Group (Years)	**Males**		**Females**	
	Number of Cases	ASR	Number of Cases	ASR
0–4	0	0	0	0
5–9	0	0	0	0
10–14	0	0	0	0
15–19	0	0	0	0
20–24	1	0.1	1	0.1
25–29	0	0	1	0.1
30–34	2	0.2	0	0
35–39	6	0.7	1	0.1
40–44	9	1.3	8	1.2
45–49	14	2.4	7	1.3
50–54	31	6.7	15	3.4
55–59	36	10	17	5.1
60–64	39	15	27	11.1
65–69	37	23.3	21	12.4
70–74	19	16.4	9	7.6
75 +	31	18.6	23	13.2

### Regional variation in pancreatic cancer incidence in Saudi Arabia

**Fig. 3:**
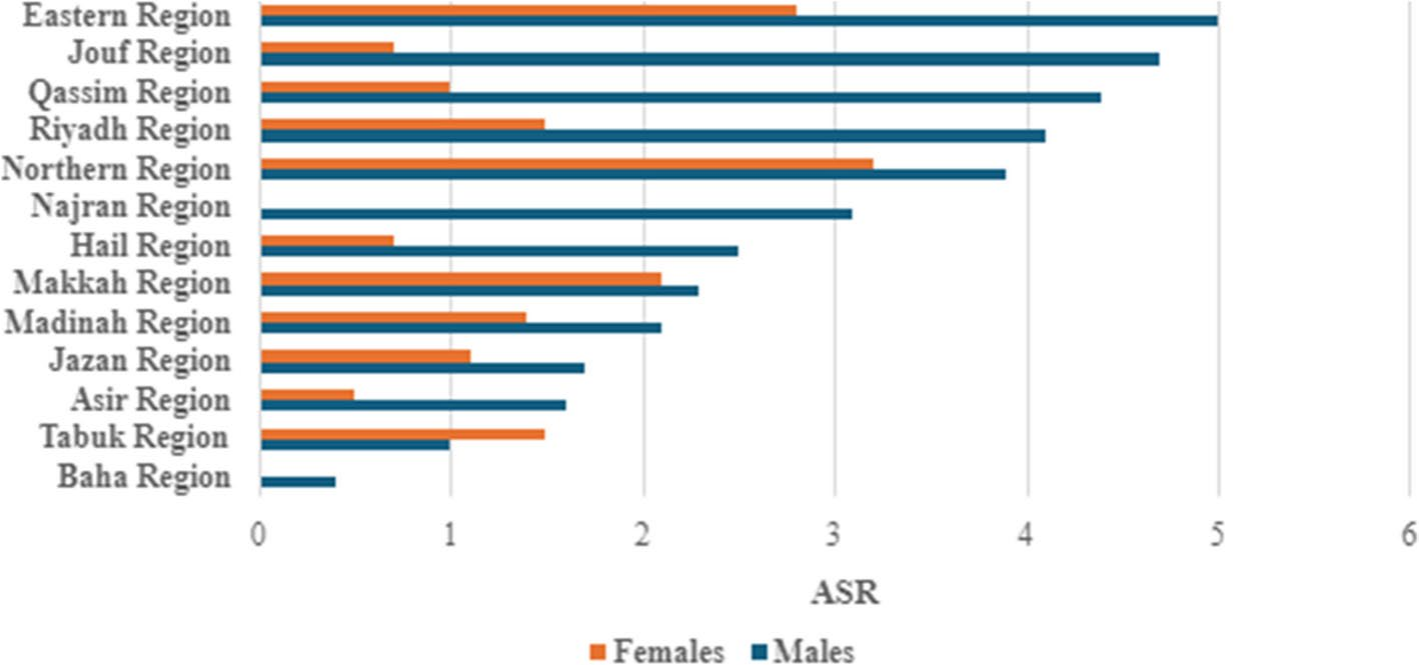
Pancreatic cancer Age Standardized Incidence Rate (ASR) in males and females in the Saudi Arabia administrative regions (Saudi Cancer Incidence Report 2020)

Saudi Arabia has 13 administrative regions. We found regional variation in pancreatic cancer incidence. Eastern region, Jouf region, and Qassim region reported the highest pancreatic cancer incidence ASRs in males (5.0, 4.7, and 4.0, respectively). In contrast, the lowest ASRs for males were reported in Baha and Tabuk regions (0.4 and 1.0, respectively). For females, the Eastern region reported the highest pancreatic cancer incidence ASR of 3.2 in contrast to no pancreatic cancer cases reported in Najran and the Baha regions (Cancer incidence report in Saudi Arabia 2020) (Table 7, Fig. 3).

**Table 7:** Pancreatic Cancer Age Standardized Incidence Rate (ASR) and number of cases in males and females in the Saudi Arabia Administrative Regions (Saudi Cancer Incidence Report 2020)

Administrative Region	Males		Females	
	Number of Pancreatic Cancer Cases	ASR Pancreatic Cancer	Number of Pancreatic Cancer Cases	ASR Pancreatic Cancer
Riyadh Region	69	4.1	35	1.5
Makkah Region	45	2.3	38	2.1
Eastern Region	43	5	27	2.8
Madinah Region	9	2.1	7	1.4
Northern Region	3	3.9	3	3.2
Qassim Region	17	4.4	4	1.0
Jazan Region	8	1.7	7	1.1
Hail Region	6	2.5	2	0.7
Najran Region	4	3.1	0	0.0
Baha Region	1	0.4	0	0.0
Asir Region	11	1.6	4	0.5
Tabuk Region	3	1.0	3	1.5
Jouf Region	7	4.7	1	0.7

### World pancreatic cancer mortality

Pancreatic cancer is the seventh leading cause of cancer death in the World, with 466,003 deaths in total. The mortality ASR for both sexes is 4.5 per 100,000 people in the World. Table 1 shows that more males than females died of pancreatic cancer, with 246,840 and 219,163 deaths, respectively. The mortality ASRs for males and females were 5.3 and 3.87, respectively.

### World regional variation in pancreatic cancer mortality

Europe and North America reported the highest mortality ASRs for pancreatic cancer, 8.8 and 7.6, respectively. The lowest mortality ASR for pancreatic cancer (2.3) was reported for African cancer patients. Pancreatic cancer ranks higher (No. 4) as a cause of cancer death in Europe and North America, while in Africa, it ranks No. 13 (Table 2).

### Pancreatic cancer mortality in the Gulf Cooperation Council countries

Mortality of pancreatic cancer in the GCC countries showed slight variation. Kuwait reported the highest mortality ASRs of 4.3, followed by Qatar and Bahrain, with 3.3 for both, 2.7 for Oman,, 2.3 for the United Arab Emirates and 2.2 for Saudi Arabia. In Kuwait, pancreatic cancer mortality ranks 6th as a cause of cancer death, while in Qatar and Oman, it ranks 9th (WHO, IARC Global Cancer Observatory website, 2020) (Table 3).

### Pancreatic cancer mortality in Saudi Arabia

Pancreatic cancer deaths in Saudi Arabia for the year 2020 were 544 deaths, according to the estimations of the WHO, IARC Global Cancer Observatory website. The 5-year survival rate was the lowest among other cancers (1.56 per 100000). Pancreatic cancer ranks 8th as a cause of cancer death (Table 8) in Saudi Arabia. The discrepancy in the number of cancer cases between the Saudi cancer registry and the IARC website is probably due to the fact that IARC provides estimations.

**Table 8:** Mortality of cancer in Saudi Arabia (WHO, IARC Global Cancer Observatory Website, 2020)

	Deaths			5-year survival		
	Number	Rank	%	Cum. Risk	Number	Prop. (per 100,000)
Liver Cancer	1105	1	8.5	0.59	1200	3.45
Breast Cancer	1095	2	8.4	0.93	13,653	92.99
Colon Cancer	1087	3	8.3	0.45	5751	16.52
Leukemia	1032	4	7.9	0.32	5726	16.45
Lung Cancer	1001	5	7.7	0.55	1442	4.14
Rectal Cancer	877	6	6.7	0.35	5167	14.84
Non-Hodgkin lymphoma	827	7	6.3	0.36	5566	15.99
Pancreatic Cancer	559	8	4.3	0.28	544	1.56
Stomach	523	9	4.0	0.25	1116	3.21
Brain, central nervous system	486	10	3.7	0.16	1876	5.39

## Discussion

The incidence and mortality rates of pancreatic cancer in Saudi Arabia have increased over time, a trend that is similar to most of the countries globally [3] for both males and females. Pancreatic cancer predominantly affects the elderly Saudi population, with over 85% of diagnoses occurring in individuals aged 50 years and older, for both males and females. Cancer in general is recognized as a disease of elder populations, this may be due to a number of factors; including the time dependent accumulation of cellular damage, epigenetic changes, in addition to the accumulative effects of environmental and life-style factors that include smoking, alcohol intake and pollution [28].

According to the 2020 Saudi cancer incidence report, pancreatic cancer ASRs were 3.0 and 1.8 for males and females, respectively [27]. These ranges are comparable to those in Gulf countries, which range between 2.4 and 4.2, but lower than the rates in Europe (ASR 7.8) and North America (ASR 8.0). The demographic structure of Saudi Arabia, with a relatively younger population compared to Europe or North America, may have contributed to the lower incidence rate in the Kingdom. Other factors that might influence the reported incidence rates include differences in genetic factors, environmental exposure, lifestyle factors, or access to healthcare. No data are available on smoking in Saudi Arabia over time. However, various studies that included selected groups of individuals reported rates of smoking in males 21.1 – 44%, and for females 0.9–13.3% indicating higher prevalence of smoking in Saudi males [29–32]. Alcohol distribution or drinking is prohibited in Saudi Arabia, rare data are available on alcohol intake in Saudi Arabia. Alcohol drinking in the East-Mediterranean countries, including Saudi Arabia, is low with a prevalence of 6.2% compared to the global estimate of 43% [33]. Alcohol intake is much higher in males than females in Saudi Arabia. 11.5% of high school male students drink alcohol [34]. Another study in Hail region reported that patients hospitalized due to alcohol consumption poisoning were mostly males [35].

The risk of pancreatic cancer occurrence increases linearly with age as well as for the other cancer types [35, 36]. Several factors may have contributed to the rise in the incidence rate of pancreatic cancer cases over time in Saudi Arabia. Advancements in healthcare services have resulted in a significant increase in life expectancy, surpassing 77 years in the past decade compared to 62 years in 1970 in Gulf countries. This region has also witnessed changes in diet and lifestyle patterns, obesity rate in this region is one of the highest in the World [35]. These changes might also have affected not only the incidence of pancreatic cancer but other types of gastrointestinal malignancies, including colorectal cancer [24]. Additionally, a previous study indicated that increasing trends in pancreatic cancer incidence in both sexes were correlated with socioeconomic development [37]. Increased use of modern diagnostic methods may have contributed to the increased detection rate of pancreatic cancer in Saudi Arabia, however; to our knowledge, no sex-variation in the accessibility to health services was reported in Saudi Arabia.

This study reveals global variations in the incidence and mortality of pancreatic cancer, with some noticeable regional and gender differences. The disease is more prevalent in Europe and North America, while Africa is the least affected. This pattern aligns with previous reports that link pancreatic cancer to HDI [4, 5]. The geographic differences in the burden of the disease might be partly explained by the variations in the prevalence of underlying risk factors for pancreatic cancer, such as diabetes, smoking, and obesity [7, 8, 11]. Furthermore, the increasing incidence rates in some countries might reflect improved access to diagnostic facilities and enhancements in the registration of pancreatic cancer.

There are significant regional variations in pancreatic cancer incidence within Saudi Arabia. The highest male pancreatic cancer age-standardized rate (ASR) for 2020 was 5 in the Eastern region, while the lowest was 0.4 in the Baha Region. Among females, the ASR for pancreatic cancer also varies across the administrative regions; the highest was 3.2 for females in the Northern region, and the lowest was 0.0 for the Najran and Baha regions females. Nevertheless, men consistently experienced higher incidences of pancreatic cancer than women in all regions of Saudi Arabia. These regional variations might be due to variations in genetic and/or environmental factors that contribute to pancreatic cancer incidence among Saudi populations in different regions. Identifying the reasons for these regional variations in Saudi Arabia necessitate further investigations.

In this study, the Riyadh region had the highest number of new cases in 2020, accounting for 30% of all pancreatic cancer cases. Obesity is believed to play a role in the increasing incidence of pancreatic cancer. The prevalence of diabetes mellitus in Saudi Arabia is rising. It has been higher in urban regions than rural parts of the country, with the highest diabetes prevalence in Riyadh [38]. Alghamdi et al*.* suggested that a high prevalence of diabetes may be associated with higher incidence of pancreatic cancer in males and females residing in Riyadh [23]. 

Our study shows variation in pancreatic cancer incidence in Saudi Arabia between males and females. Sex-related variations are common findings in the incidence of many cancer types [24], which may be attributed to a combination of factors that may include sex hormone differences and genetic and epigenetic factors [13]. Sex-variation in the incidence of pancreatic cancer may be explained partially by different rate of smoking between males and females. Almost all published studies reported higher rates of smoking in Saudi males compared to Saudi females [29–31, 39]. Pancreatic cancer mortality rates in Saudi Arabia show an increasing trend both in males and females. The survival rate of pancreatic cancer patients in Saudi Arabia is the lowest compared to other types of cancer, due to delayed disease detection and limited effectiveness of therapies, the survival for pancreatic cancer remains poor [22].

Worldwide, the number of deaths from pancreatic cancer (466003) was almost the same as the number of new cases (495773), indicating the high mortality of the disease. The survival rates for pancreatic cancer patients vary little between HICs and LMICs [40], and their mortality is still largely determined by their incidence [1]. Even in HICs, only about 10% are still alive five years after diagnosis.

In conclusion, this study provides valuable insights into the incidence and mortality of pancreatic cancer in Saudi Arabia. The increasing trend of pancreatic cancer incidence underscores the need for ongoing research and public health interventions to understand and address this disease. Regional variations exist within the country, and the higher incidence in older age groups and among males indicate potential risk factors that are waiting to be investigated and identified in order to build robust strategies for pancreatic cancer prevention and early detection in Saudi Arabia.

## Data Availability

The datasets used and/or analyzed during the current study available from the corresponding author on reasonable request. Data were collected from the following two publicly available data sources:
